# Efficacious switching from subcutaneous to intravenous tocilizumab in patients with non-infectious non-anterior uveitis

**DOI:** 10.1186/s12348-023-00336-3

**Published:** 2023-07-18

**Authors:** Mathilde Leclercq, Paul Goupillou, Hélène Gomez, Marc Muraine, Ygal Benhamou, Nicolas Girszyn, Julie Gueudry

**Affiliations:** 1 CHU Rouen, Internal Medicine Department, CHU Charles Nicolle, 1, Rue de Germont, 76000 Rouen, France; 2 Ophthalmology Department, CHU Rouen, 76000 Rouen, France; 3 EA7510, UFR Santé, Rouen University, 76000 Rouen, France

**Keywords:** Uveitis, Tocilizumab, Body weight, Subcutaneous, Drug monitoring

## Abstract

**Purpose:**

The efficacy of tocilizumab in refractory chronic noninfectious uveitis has been previously reported, but no data comparing intravenous and subcutaneous tocilizumab in uveitis are available.

**Results:**

We report a case series of patients with chronic noninfectious uveitis with incomplete efficacy of subcutaneous tocilizumab, improved after switching to intravenous routes. Improvement of visual acuity was observed with intravenous tocilizumab for all patients. Half of the patients could stop corticosteroids. Rapid efficacy of intravenous tocilizumab was observed, between 2 and 3 months.

**Conclusion:**

In uveitis, tocilizumab administration could be optimized by a switching from a subcutaneous to an intravenous administration route.

## Introduction

Tocilizumab is an anti-interleukin-6 (IL6) receptor antibody approved for the treatment of rheumatoid arthritis (RA), juvenile idiopathic arthritis (JIA) and giant cell arteritis, among other conditions. It was first used intravenously at a dose of 8 mg/kg at 4-week intervals and then subcutaneously at a fixed dose of 162 mg per week, not adapted to body weight. The efficacy and safety of subcutaneous versus intravenous tocilizumab was demonstrated in the SUMMACTA trial, a prospective study of 1262 patients with RA [1]. The efficacy of tocilizumab in refractory chronic noninfectious (CNI) uveitis has been previously reported in retrospective studies; tocilizumab was administered intravenously, in 91% to 100% of cases [2, 3]. Intravenous tocilizumab may be useful in refractory macular edema associated with CNI uveitis [4]. No data comparing intravenous and subcutaneous administration in non-infectious non-anterior uveitis are available. The question of the efficacy of subcutaneous tocilizumab in uveitis is currently a hot topic, and some authors have suggested that subcutaneous tocilizumab is less effective [5].

The interest of monitoring serum drug levels and antidrug antibodies has been well documented with anti-TNF-α therapy in inflammatory diseases [6]. However, there are few data in the literature on the value of such monitoring for tocilizumab. The therapeutic target concentration of tocilizumab has been evaluated in RA and seems to be 10 µg/ml [7].

Here we report a case series of patients with CNI uveitis with incomplete efficacy of subcutaneous tocilizumab, improved after switching to intravenous routes. Three out of the four patients had a body mass index (BMI) of > 35 kg/m^2^ and two patients benefited of drug monitoring.

## Case reports

### Case n°1

A 35-year-old woman was diagnosed with birdshot chorioretinopathy in 2014 (Table 1). The patient's weight was 98 kg, with a body mass index (BMI) of 37.8 kg/m2. She was first treated with methylprednisolone pulses associated with interferon α2a (Roferon®, Roche laboratory, France) at a dose of 3 million units/injection 3 times a week. Macular edema relapsed in both eyes at a dose of 60 mg/day of corticosteroid and infliximab (Remicade®, MSD laboratory, France) was introduced 3 months later. Infliximab (5 mg/kg/6 weeks) was also ineffective to control macular edema. In April 2015, tocilizumab was introduced intravenously at a dose of 8 mg/kg/4 weeks (Roactemra®, Roche laboratory, France) with prednisone (30 mg/day). In July 2017, tocilizumab was switched subcutaneously (162 mg/week) due to disease control, with resolution of macular edema. The corticosteroid dose was 10 mg/day. Four months later, a relapse occurred; visual acuity remained unchanged but papillary edema and a visual field defect appeared in the left eye (LE). Tocilizumab (Roactemra®) was re-introduced intravenously (8 mg/kg/4 weeks). Three months later, an improvement was observed with papillary edema resolution. At the end of follow-up, i.e. December 2022, visual acuity was 20/20 in the right eye (RE) and 20/200 in LE. Corticosteroids were stopped at 10 months and intravenous tocilizumab was continued as monotherapy. Tocilizumab injections were spaced every 6 weeks since February 2022 without relapse. No side effect related to tocilizumab occurred during follow-up.

**Table 1:** Demographics and clinical characteristics of patients with lack of efficacy of subcutaneous tocilizumab

	**Date of diagnosis**	**Etiology**	**Previous treatment**	**BMI (kg/m**^**2**^**)**	**Cortico-steroid dose at time of SC TCZ relapse**	**Time to relapse**	**Relapse characteristics**	**Time to efficacy**	**End of follow up after IV TCZ**	**Cortico-steroid dose at the end of follow-up**
Case n°1	2014	Birdshot	Methyl-prednisolone, interferon α2a, infliximab, tocilizumab IV	37.8	10 mg	4 months	Papillary edema, scotoma LE	3 months	61 months	0 mg
Case n°2	2017	Birdshot	Prednisone, MMF, adalimumab	27	10 mg	6 months	Papillary edema, scotoma OU	3 months	12 months	8 mg
Case n°3	2019	Behçet	Methyl-prednisolone, infliximab, adalimumab	36.4	20 mg	3 months	Macular edema OU	3 months	31 months	0 mg
Case n°4	2017	Idiopathic	Prednisone, MTX	46.3	10 mg	5 months	Retinal vasculitis OU	2 months	18 months	5 mg

*SC TCZ* Subcutaneous tocilizumab, *IV* Intravenous, *LE* Left eye, *MMF* Mycophenolate mofetil, *OU* Both eyes, *MTX* Methotrexate

### Case n°2

A 61-year-old man was diagnosed with birdshot chorioretinopathy in October 2017 (Table 1). The patient’s weight was 77 kg, with a BMI of 27 kg/m^2^. He was initially treated with prednisone (1 mg/kg) and mycophenolate mofetil 2 g/day (Cellcept®, Roche laboratory, France). In February 2019, a macular edema relapse occurred in LE and adalimumab (Humira®, AbbVie laboratory, France) was introduced (40 mg/2 weeks). In July 2020, at a dose of 10 mg/day of prednisone, the visual field defect worsened, in a context of steroid induced glaucoma. A switch to subcutaneous tocilizumab (Roactemra®, 162 mg/week) was made. Six months later, a decrease in visual acuity, papillary edema and a visual field defect occurred in both eyes. Five days after injection, tocilizumab plasma residual was 11 µg/ml. There were no anti-tocilizumab antibodies. Intravenous tocilizumab (Roactemra®, 8 mg/kg/4 weeks) was introduced. Three months later, visual acuity improved to 20/20 in RE and 20/25 in LE, with regression of papillary edema. In March 2022, after 12 months of follow-up, visual acuity was 20/20 in both eyes. The corticosteroid dose was 8 mg/day. No side effect related to tocilizumab occurred during follow-up.

### Case n°3

**Fig. 1:**
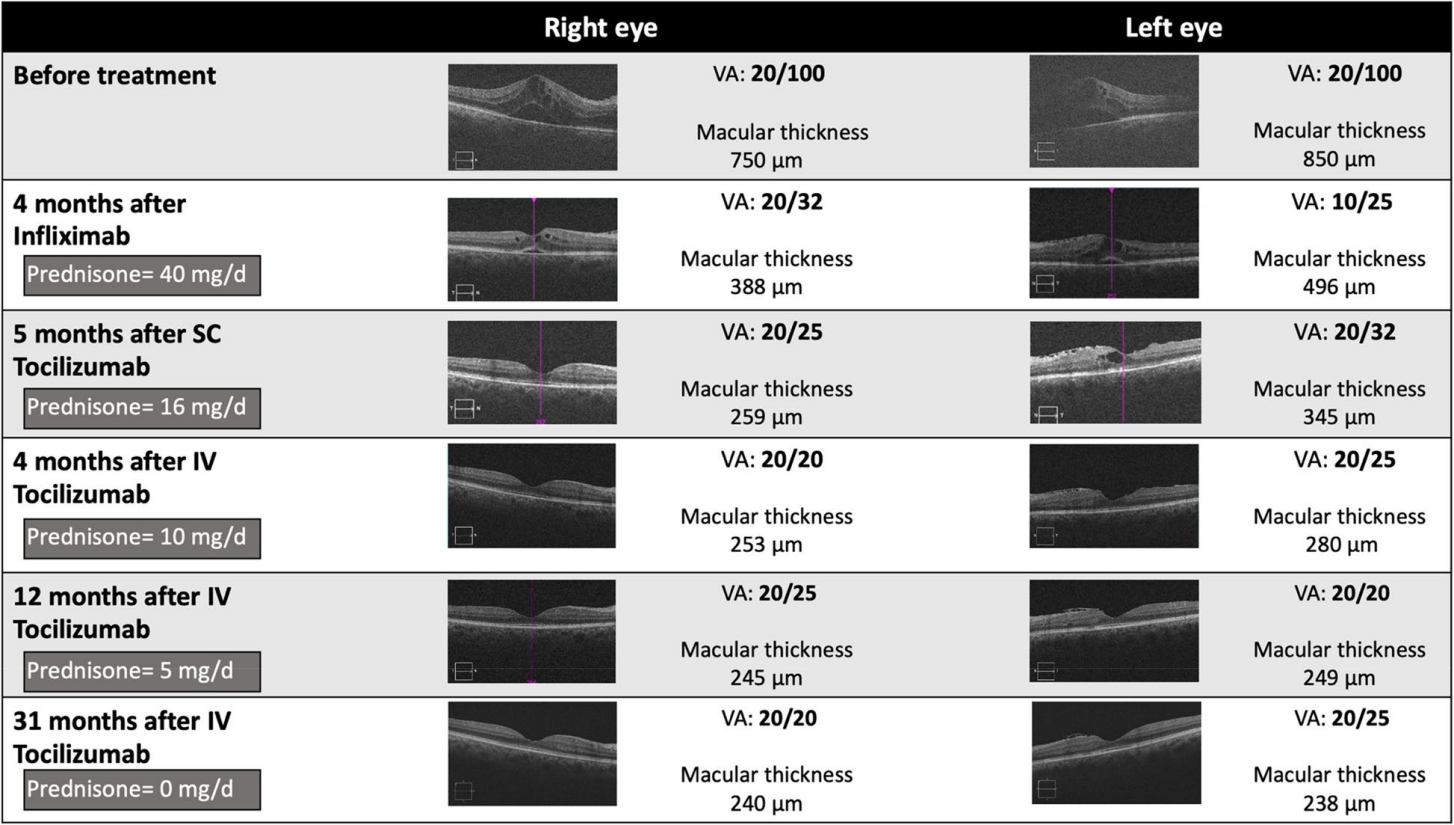
Evolution of patient 3 visual acuity and macular edema on infliximab, subcutaneous (SC) tocilizumab and after the switch for intravenous (IV) tocilizumab. D: day, VA: visual acuity

A 48-year-old woman was diagnosed with Behçet's disease in May 2019 (Table 1). The patient's weight was 104 kg, with a BMI of 36.4 kg/m^2^. Initial visual acuity was 20/100 with macular edema in both eyes. She was treated with methylprednisolone pulses and infliximab (Remicade®) at 5 mg/kg at weeks 0, 2, 6 and then every 6 weeks. In September 2019, there was no improvement of macular edema, despite a high corticosteroid dose (40 mg/day). Adalimumab (Humira®) was introduced (40 mg/2 weeks). In January 2020, tocilizumab (Roactemra®) was introduced subcutaneously (162 mg/week) because of macular edema relapse in LE. The corticosteroid dose was 20 mg/day. However, 3 months after treatment beginning, there was no improvement of macular edema or visual acuity. Tocilizumab was switched to an intravenous route. Three months later, there was an improvement of macular edema and visual acuity (20/20 in RE and 20/25 in LE). The corticosteroid dose decreased to 10 mg/day. Twelve months after the treatment switch, visual acuity was 20/25 in RE and 20/20 in LE, without any ocular inflammation or macular edema and the corticosteroid dose was 5 mg/day. At the end of follow-up, i.e. 31 months after treatment beginning, visual acuity still improved with 20/20 in RE and 20/25 in LE. Interestingly, tocilizumab injections were spaced every 6 weeks since April 2022 and corticosteroids were stopped in September 2021. Figure 1 represents the evolution of this patient on infliximab, subcutaneous tocilizumab and after the switch for intravenous tocilizumab. No side effect related to tocilizumab occurred during follow-up.

### Case n°4

A 40-year-old woman was diagnosed with posterior idiopathic uveitis in April 2017, initially treated with corticosteroid eye drops (Table 1). The patient's weight was 117 kg, with a BMI of 46.3 kg/m^2^. In August 2019, because of retinal vasculitis, a systemic corticosteroid, and methotrexate at 0.3 mg/kg/week (Metoject®, Medac laboratory, France) were introduced. Fourteen months later, retinal vasculitis reappeared on fluoresceine angiography. The corticosteroid dose was increased to 40 mg/day and tocilizumab (Roactemra®) (162 mg/week sub-cutaneous) was introduced. Five months later, there was no improvement of intra-ocular inflammation. The corticosteroid dose was 10 mg/day. One week after injection, tocilizumab plasma residual was 10.5 µg/ml. There were no anti-tocilizumab antibodies. The corticosteroid dose was increased to 20 mg/day and intravenous tocilizumab was introduced. Two months after, vasculitis improved on angiography. After 6 months of treatment, retinal vasculitis was still improved, and visual acuity was 20/20 in both eyes. The corticosteroid dose was decreased to 10 mg/day. In February 2022, secondary to persistent ocular inflammation, tocilizumab injections were proposed every 3 weeks, which improve inflammation and allow a decrease of corticosteroid dose to 5 mg/day. No side effect related to tocilizumab occurred during follow-up.

## Discussion

We report 4 cases of CNI uveitis with lack of efficacy of subcutaneous tocilizumab (Table 1), which improved after switching administration route. An improvement of visual acuity with intravenous tocilizumab was observed for all patients. Moreover, corticosteroids could be stopped for two patients. Rapid efficacy of intravenous tocilizumab was observed, between 2 and 3 months. No specific side effect was reported. Interestingly, three out of the four patients had a BMI > 35 kg/m^2^.

Two prospective trials studied the efficacy of tocilizumab in CNI uveitis. STOP-uveitis included 37 patients with non-anterior uveitis: 18 patients received 4 mg/kg/4 weeks of tocilizumab and 19 patients received 8 mg/kg/4 weeks [4]. Active uveitis was confirmed on vitreous haze assessment. The authors observed a significant improvement in visual acuity, a decrease in vitreous haze and a reduction of central foveolar thickness. There was no significant difference between the two doses of tocilizumab, and no difference between naïve patients and patients previously treated with anti-TNF-α. Tocilizumab, administered subcutaneously, was studied in JIA in the APTITUDE study [8]. This was a multicenter single-arm study including 21 patients with active uveitis, refractory to anti-TNF-α agents. One third of the patients stopped the treatment before the first 3 months because of inefficacy. The study did not meet the prespecified criterion at 12 weeks to justify a phase 3 trial, suggesting less efficacy of subcutaneous tocilizumab in CNI uveitis.

Only half of the patients could stop corticosteroids, the other two patients had to maintain corticosteroid doses greater than or equal to 5 mg/day. In addition, patient n°4 had to bring the tocilizumab injections closer due to a decrease in the effectiveness of the treatment. We still concluded that the treatment was effective. Indeed, the patients we present are multi-refractory patients, with failure of several lines of treatment, with limited therapeutic possibilities.

Abdallah et *al*. found no difference in terms of efficacy or immunogenicity between intravenous and subcutaneous tocilizumab for 1759 patients with RA [9]. Because subcutaneous tocilizumab was administered at a fixed dose, independent of body weight, the correlation between efficacy and body weight was studied [7, 9]. Tocilizumab concentration was lower for patients with a body weight > 100 kg, i.e. a maximum observed concentration of 69.7 µg/ml for a body weight < 60 kg versus a maximum observed concentration of 26.9 µg/ml for a body weight ≥ 100 kg, and was inversely correlated with body weight. In Arad's study [7], including 100 patients with RA treated with subcutaneous tocilizumab at a dose of 162 mg/week, body weight was inversely correlated with disease improvement. Abdallah et *al*. reported a target concentration of tocilizumab in RA of 10 µg/ml [9]. There were no anti-drug antibodies in this study. In our study, cases n°2 and n°4 did not have anti-drug antibodies and had a tocilizumab plasma residual level of > 10 µg/ml, with a body weight > 100 kg. No study has focused on the correlation between tocilizumab plasma residual and intraocular inflammation control. The ocular immunological environment may evolve independently of the peripheral circulation, explaining an incomplete correlation. Interestingly, three out of four patients had a BMI > 35 kg/m^2^, suggesting that, in this population, intravenous tocilizumab might be used in cases of CNI uveitis, for better bioavailability. We did not suspect medication non-adherence in our patients.

Quesada-Masachs et *al*. [10] also reported the inefficacy of subcutaneous tocilizumab in chronic anterior uveitis in JIA, although all patients were previously controlled with intravenous tocilizumab; switching to subcutaneous tocilizumab was done for patient comfort. Treatment management after relapse was not described. Conversely, in our study, three of the four patients were first treated with subcutaneous tocilizumab.

Interestingly, in CNI uveitis, a similar difference between subcutaneous and intravenous administration route has been described with secukinumab, an anti-IL17 agent. Dick et *al*. [11] reported the inefficacy of subcutaneous secukinumab in three randomized controlled trials. However, Letko et *al*. [12] showed a superiority of intravenous versus subcutaneous secukinumab to control intra-ocular inflammation with corticosteroid sparing effect in 33 patients.

Our study has some limitations. This is a retrospective study, with a small population and an inhomogeneous follow-up of all patients. Moreover, the therapeutic strategies chosen may be questionable, particularly the absence of an increase in the dosage of infliximab, explained by the prescription practices.

To summarize, in CNI uveitis, we suggest that before considering tocilizumab failure, therapeutic optimization could be obtained by a switching from a subcutaneous to an intravenous administration route, allowing corticosteroid sparing effect.

## Data Availability

Not applicable.
